# Do We Have a Match? Assessing the Role of Community in Coworking Spaces Based on a Person-Environment Fit Framework

**DOI:** 10.3389/fpsyg.2021.620794

**Published:** 2021-02-09

**Authors:** Eileen Lashani, Hannes Zacher

**Affiliations:** Department of Work and Organizational Psychology, Institute of Psychology – Wilhelm Wundt, Leipzig University, Leipzig, Germany

**Keywords:** coworking, coworking space, community, person-environment fit, needs-supplies fit

## Abstract

As working arrangements become more flexible and many people work remotely, the risk of social isolation rises. Coworking spaces try to prevent this by offering not only a workplace, but also a community. Adopting a person-environment fit perspective, we examined how the congruence between workers' needs and supplies by coworking spaces relate to job satisfaction and intent to leave. We identified five needs (i.e., community, collaboration, amenities, location, and cost), of which community was expected to be the central need. An online questionnaire was distributed among coworkers in Germany and Austria, resulting in a sample of 181 coworkers. Results showed that needs-supplies fit regarding community was related to job satisfaction and intent to leave in coworking spaces. Findings for the other needs, however, did not show that congruence is associated with outcomes. Overall, the findings highlight the importance of community fit in coworking and offer insights for workers and entrepreneurs in this area.

## Introduction

Advances in technology and digitalization allow workers more flexibility in where they carry out their tasks. There has been a shift away from traditional office work toward alternative arrangements, such as telework or independent freelance work (Osnowitz, 2010; Kuhn, 2016; Katz and Krueger, 2019). Especially knowledge workers (people who “think for a living;” Davenport, 2005), for instance data analysts, lawyers, engineers, or web designers, benefit from this development. In the United States, the number of people regularly working from home grew by 140% since 2005 (Global Workplace Analytics, 2017).

As attractive as the promises of autonomy, flexibility, and independence seem, remote work also has downsides. Studies show that remote workers report feelings of social isolation, self-motivation problems, and a lack of recognition and support (Cooper and Kurland, 2002; Spinuzzi, 2012; Johns and Gratton, 2013). To combat these feelings, alternatives to the working from home have been introduced as so called “third places” (i.e., informal meeting places between the domestic home and the productive workplace; Oldenburg, 1989), such as coffee shops or libraries. Yet, they seem unable to offer adequate working conditions or the social network that remote workers often hope to find there (Hampton and Gupta, 2008; Spinuzzi, 2012). This has led to the rise of coworking spaces. DeGuzman and Tang (2011) refer to coworkers as “a diverse group of people who don't necessarily work for the same company or on the same project, working alongside each other, sharing the working space and resources” (p. 22). Garrett et al. (2017), added another central aspect, namely striving for a community, to the definition: “Coworkers pay a monthly fee to share a space with other freelance/remote workers with an explicit purpose of social belonging” (p. 822).

Since the foundation of the first coworking space in San Francisco in 2005, there has been an exceptional growth of the coworking movement. From 8,900 coworking spaces worldwide in 2015, the number increased to an estimated 26,300 in 2020 (Deskmag, 2019). Within only 3 years, the number of coworkers more than tripled to 1,650,000 in 2018 (Deskmag, 2019). At the same time, it is surprising that coworking remains an understudied phenomenon in the literature (for two exceptions, see Moriset, 2013; Gerdenitsch et al., 2016). With the current study, we add to this literature by examining the motives for coworking. While most former studies collected coworkers' motives in qualitative case studies, we investigate the impact of the most prominent motives or needs on work related outcomes in a comprehensive quantitative approach.

People differ in their needs when looking for a job or a place to work (Nakai et al., 2011). Some people might have a stronger need for autonomy or responsibility in their job, whereas others might value flexible working hours or payment more highly. Organizations and workplaces also differ in what they supply to satisfy workers' needs. A common approach to examine these issues is the person-environment fit (PE fit) framework (e.g., Edwards et al., 1998), specifically the needs-supplies fit (NS fit) dimension of it. The central assumption of the theory is that a good fit, that is, a congruence between needs and supplies, leads to positive worker outcomes, such as job attitudes. The most common needs identified in previous research on coworking spaces are community, collaboration, amenities, location, and cost (e.g., Spinuzzi, 2012; Bilandzic and Foth, 2013; Merkel, 2015; Deskmag, 2017). This study focusses on the extent to which the supply of these needs influences coworkers' job satisfaction and intent to leave. The theoretical contribution of this study consists in testing the traditional framework of PE fit in modern alternative work environments. Thereby, the results will add to the ongoing discussion about the conceptualization of NS fit as exact congruence or general compatibility.

From a practical point of view, the assessment of PE fit yields implications for both coworking space operators and users. Given that the perceived fit of a person and an organization is the base for a job application (Judge and Cable, 1997), one can assume that the perceived fit with the profile of a coworking space is also vitally important when signing up for a membership. Therefore, the results of the present study will help operators to tackle their current number one challenge: attracting new members (Deskmag, 2019). For users, the findings can be used for guidance, when deciding among the constantly increasing number of coworking spaces.

**Figure 1:**
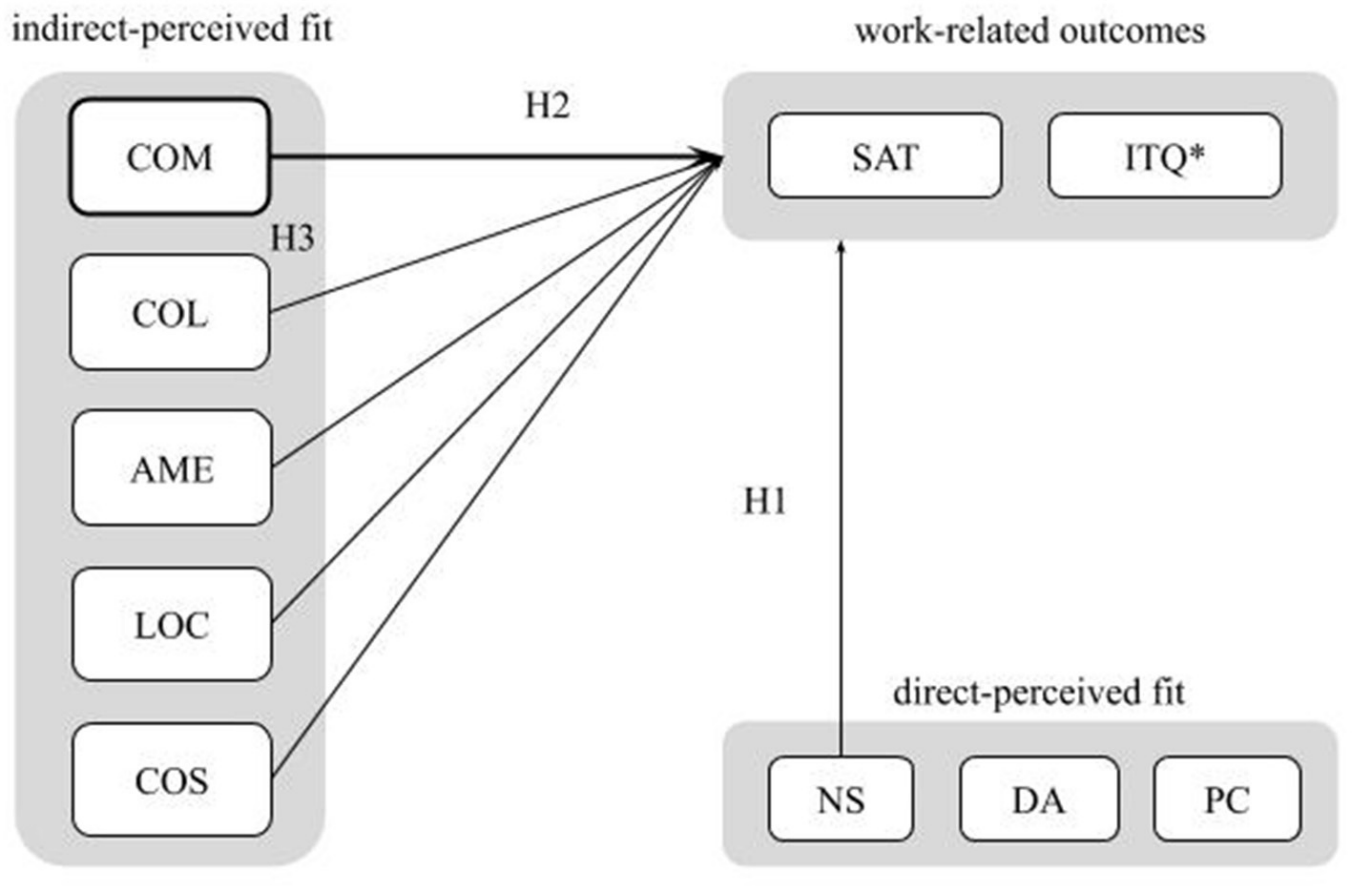
Conceptual model. COM, community; COL, collaboration; AME, amenities; LOC, location; COS, cost; SAT, job satisfaction; ITQ, intent to quit; *, negative outcome; NS, needs-supplies fit; DA, demands-abilities fit; PC, person-coworking space fit (supplementary fit).

A conceptual model of this study which summarizes all hypotheses is represented in Figure 1.

## Theory and Hypotheses

### Coworking and Needs Supplies Fit

To apply the theoretical PE fit framework to coworking, it is important to understand in which context the coworking phenomenon arose. Beginning in the 1980s, technical devices such as home computers, internet, and e-mailing enabled a previously unknown amount of flexibility and mobility for knowledge workers and employers (Osnowitz, 2010; Johns and Gratton, 2013). Taking advantage of the new possibilities, more and more freelancers and teleworkers entered the labor market. The number of people pursuing a self-employed, freelance, or telework job has grown significantly and is still rising (Kuhn, 2016; Global Workplace Analytics, 2017; Katz and Krueger, 2019). As technology kept developing and expanding, the globalization of teams and whole companies became possible. This led to a further increase in options, time- and geographical flexibility. By 2013, “One in five Americans work from home” (Rapoza, 2013, p. 1). Apart from the many advantages the new work arrangements hold, it became clear that there is also a downside to the modern way of work. Remote workers often report feelings of isolation, lack of social interaction, loss of team feedback, reduced motivation, and less perceived support (Vega and Brennan, 2000; Ward and Shabha, 2001). It seemed that there was hardly a good alternative to the confined traditional office work or the isolation when working from home. Working from so called “third places” (Oldenburg, 1989), such as cafés or libraries, did not turn out as an adequate solution either. It has been shown that those places do not offer the social interaction that solo workers aim for (Hampton and Gupta, 2008), nor do they supply an appropriate long-term work environment (Spinuzzi, 2012). Out of this dilemma, a new movement evolved. “The third wave of virtual work,” as it was called by Johns and Gratton (2013), brought up new working models, which are supposed to provide a community and shared space and thereby foster collaboration among individuals. The most prominent of these working arrangements is coworking.

DeGuzman and Tang (2011, p. 22) refer to coworkers as “a diverse group of people who don't necessarily work for the same company or on the same project, working alongside each other, sharing the working space and resources.” The concept of coworking involves the coworker or user (i.e., the working individual) and the coworking space (i.e., the workplace) (Uda, 2013). The coworker usually pays a regular fee for physically sharing the workplace, using its amenities (e.g., Wi-Fi, computers, desks, conference rooms, and cafés) and participating in activities or events organized by the coworking space (e.g., talks, workshops, launch parties, game nights, and joint breakfasts). In contrast to rental offices, which aim at providing a functional place to work, coworking spaces create a productive work atmosphere and a membership in a social community.

Theories of PE fit are one of the most investigated themes in industrial and organizational psychology (Kristof-Brown et al., 2005). Broadly defined, PE fit describes the match between characteristics of individuals and their work environment (Greguras and Diefendorff, 2009). It has been conceptualized in many ways, but there are three key assumptions that all perspectives on PE fit share: People seek out and create environments where they can act consistent with their traits; a good fit between person and environment leads to positive work-related outcomes; and PE fit is understood as a reciprocal process in which person and environment mutually influence each other (Su et al., 2015).

To integrate the diverse approaches, Edwards and Shipp (2007) determined three perspectives on PE fit. Generally, the concept of fit encompasses two types of compatibility. One is based on similarity (i.e., supplementary fit) and the other is based on mutual completion (i.e., complementary fit) (Muchinsky and Monahan, 1987). Complementary fit can be viewed from both perspectives; that of the person and that of the environment.

From the environment-perspective, demands-abilities fit (DA fit) is high when the person is able to meet the demands that the environment imposes on them. The person, on the other hand, has needs from the organization, as well. This perspective on complementary fit is called needs-supplies fit (NS fit). NS fit is high, when the environment supplies the resources, that the person needs. Of these three types of fit (supplementary fit, DA fit, and NS fit) we focus on NS fit in this study for the following reasons.

In the past, many fit studies have focused on supplementary fit and DA fit when investigating job outcomes (Cable and DeRue, 2002). The focus of coworking spaces, however, lays on the person-perspective. It has become apparent, that the characteristics of coworking spaces and their users differ considerably from traditional work arrangements. Whereas, traditionally the properties of the environment were primarily in the hands of the employing organization, they now lie with the job holder. Due to their great flexibility, many self-employed, freelance and remote workers have the privilege to choose their workplace themselves. The perspective of the individual has therefore become more important than ever before. Workers, who have a variety of options (e.g., home office, different rental offices and coworking spaces) chose to work in the coworking space, that best matches their needs. In consequence to this change toward more freedom of choice, it is also necessary to change the focus of PE fit research. We therefore assume complementary NS fit to have the highest relevance in coworking spaces compared to other perspectives of PE fit.

Having clarified the focus of this study, the assumed outcomes of NS fit can be specified. Originally, PE fit was investigated in the context of stress theory (Edwards et al., 1998), but has been shown to influence many other central work-related outcomes. For this study, we decided to consider the most relevant positive and negative attitudes in the context of work, which are job satisfaction and intent to quit.

Job satisfaction is the affective or emotional response that results from the cognitive comparison of actual and desired aspects of the job (Cranny et al., 1992). NS fit results, if the needs of a person and the supplies an environment offers are congruent. It is therefore directly linked to job satisfaction as an affective response to the level of fit (Edwards and Shipp, 2007). In addition to this direct association, Greguras and Diefendorff (2009) propose a model, which includes another indirect link. Using self-determination theory, they explain how the fulfillment of higher-order psychological needs acts as a mediator between PE fit and job satisfaction. In their study, they showed that a high level of fit led to high levels of perceived autonomy, competence, and relatedness. This psychological need fulfillment, in turn, led to an increased satisfaction of the participants. Applied to NS fit, for instance, the supply of needed characteristics in the work environment can lead to higher efficacy at work, resulting in higher perceived competence, leading to more job satisfaction. Supporting these theoretical assumptions, meta-analytical findings have shown that NS fit, compared to other types of fit, is the strongest predictor for job satisfaction (Kristof-Brown et al., 2005).

Job satisfaction, in turn, is considered the strongest predictor of turnover intentions (Elangovan, 2001). Consequently a high level of NS fit has been meta-analytically associated with lower turnover intentions (Kristof-Brown et al., 2005). These empirical findings are also supported by the theory of work adjustment (Dawis and Lofquist, 1984), which is derived from PE fit theory. It states that a person will try to adjust their work environment or their expectations toward it to perceive their needs and the supplies of the environment as congruent. In line with the theory of work adjustment, high perceived fit motivates the person to maintain their situation. Misfit, on the other hand, motivates the person to modify it. In this context, the most evident way to do so, is by changing the workplace.

*Hypothesis 1*: NS fit is positively associated with (a) job satisfaction and negatively associated with (b) intent to quit.

### Content Dimensions of Needs-Supplies Fit in Coworking Spaces

As Campbell et al. (1970) noted, PE fit theory is a pure process theory that does not specify the actual content dimensions in which fit should occur. Two questions we try to answer with this study are: What do coworkers need? And which needs have the most impact on work-related attitudes and behavior?

The first and most apparent need coworkers have is the need for a community. Some authors even include “the express purpose of being part of a community” (e.g., Garrett et al., 2017, p. 821) in their definition of coworking. Creating a community happens to be one of the reasons the coworking phenomenon emerged, in order to cure the side-effects of virtual worker isolation (Johns and Gratton, 2013). But what is the reason for the detrimental effects of isolation on attitudinal and work-related outcomes (Cooper and Kurland, 2002; Golden et al., 2008)? The importance of social belonging has been long discussed in motivational theories. What many of these theories have in common, is that humans strive for social belonging and affiliation (Murray, 1938; Baumeister and Leary, 1995). Applied to the work context, theories which feature the special role of community and social support in the process of task performance and stress have developed. For example, Karasek and Theorell (1990) assume in their job-strain-model, that a higher level of perceived social support decreases the negative effects of high demands and low decision latitude on stress. This positive effect of social support on work outcomes has also been found in coworking spaces (Gerdenitsch et al., 2016). In general, coworking helped users to increase the size of their social circle and decreasing their sense of isolation (Waber et al., 2014). It appears that coworking is more than just “working alone together” (Spinuzzi, 2012). As a coworker from Singapore Impact hub states “It is not about a business transaction, it is about social support... needing and being needed.” (Castilho and Quandt, 2017, p. 32).

Following the importance of social interaction, another aspect seems inevitable to mention. Some coworker's needs go beyond the mere presence of others. They do not only want to have a nice chat by the coffee machine every now and then, but they actively want to exchange their knowledge and work together with others. Especially for one part of the target group of coworking spaces, i.e., freelancers and self-employed, networking is a vital component of their work (Süß and Becker, 2013). The branch diversity and open setting in coworking spaces offer perfect conditions for knowledge exchange, social learning and collaborations (Waber et al., 2014). In contrast to serviced offices, the degree of social collaboration is much higher in coworking spaces (Waters-Lynch et al., 2016).

Despite community and collaboration possibilities, coworking spaces still are a place for individuals to work on their own projects. The quality of the amenities the coworking space offers therefore is one of the main motives why people use the space (Bilandzic and Foth, 2013). While some individuals may only need a desk and Wi-Fi, others might require meeting rooms or more sophisticated hard- and software. Amenities can also include more recreational-focused equipment, such as cafés, gardens, or even indoor soccer fields. Space design is an important factor for many coworkers, too (Spinuzzi, 2012).

From the way a space is equipped, we now come to where it is located. Some users have reported that the distance from home is one of the most decisive factors (Deskmag, 2017). Others focus on the distance to the city center, transport connections, or other specific facilities. Hence, a convenient location of the coworking space is another need to consider here.

One last fit dimension that cannot be left out is adequate cost. The price range of coworking space memberships, as well as the willingness of users to pay varies considerably (Miller et al., 2016). For some people, cost can even be the determinant factor when choosing to cowork, since the memberships tend to be a cheaper alternative to rental offices.

We purposefully chose these five rather broad content dimensions, because they contain need facets, that have repeatedly been mentioned in qualitative studies and surveys about coworking spaces (e.g., Spinuzzi, 2012; Deskmag, 2017).

*Hypothesis 2:* NS fit in coworking spaces with regard to community, collaboration, amenities, location and cost positively relates to job satisfaction and negatively to intent to quit.

*Hypothesis 3:* The need for community has (a) the highest subjective importance in coworking spaces and (b) best predicts work-related outcomes compared to the other needs (i.e., collaboration, amenities, location, and cost).

## Methods

### Participants and Procedure

A list of 365 coworking spaces in Germany and 87 in Austria was compiled. We only included coworking spaces, that offered rentable workspace and hosted a minimum of five people. This led to exclusion of shared or rental offices, business centers, and coworking spaces within organizations. We first contacted the resulting 452 coworking spaces via standardized e-mails. As an incentive for sharing our study among the coworkers, we offered the space managers an individual report of their user's attitudes toward the coworking space. Due to very low response rates, we conducted follow-up phone calls, sent out reminder e-mails and approached selected coworking spaces in person. Additionally, we shared our study-link on social media (e.g., Facebook) and coworking network pages. Our recruitment efforts led to the participation of *N* = 181 German speaking coworkers from 83 different coworking spaces. The study was conducted according to the guidelines of the German Psychological Society and was voluntary and anonymous. All procedures performed in this study were in accordance with the ethical standards of the 1964 Helsinki Declaration and its later amendments or comparable ethical standards.

One hundred fifteen participants were male (63.54%), 64 female (35.36%) and two diverse (1.10%). Age ranged from 20 to 66 years (M = 35.73 years, SD = 10.17). The participants worked in a broad range of professions, most of which fall into the category of knowledge work (e.g., software engineers, web developers, graphic designers, and consultants). We asked the participants to select the industry they would assign their job to, based on the International Standard Industrial Classification (ISIC) of the United Nations Statistical Division (2008). The most represented branch was information and communication (38.67%), followed by professional, scientific and technical activities (17.68%), other service activities (13.26%), administrative and support service activities (6.08%), and arts, entertainment and recreation (4.42%). 13.26% assigned their work to other branches or stated that they could not assign their job to one of the presented classifications (6.63%). Concerning the contract type of the participants, 95 were self-employed (52.49%), 55 full-time employed (30.39%), 17 part-time employed (9.39%), six were doing an internship (3.31%), three were mini-jobbers (1.66%) and five stated they had another type of work-relation (2.76%).

The duration of the membership in the coworking space ranged from half a month to 8 years (*M* = 16.48 months, *SD* = 16.79). On average, users spent between three and 80 working hours (*M* = 28.51 h, *SD* = 14.38) and between zero and 50 leisure hours (*M* = 2.33 h, *SD* = 4.87) in the coworking space each week. They usually shared a room with zero to 65 people (*M* = 7.59, *SD* = 9.01). Eighty-nine coworkers paid the membership fee themselves (49.17%), 80 had it covered by their employer (44.20%), and twelve coworkers had other payment agreements (6.63%), e.g., they did not pay because they work for the coworking space. Most users agreed that they worked in the coworking space voluntarily (*M* = 4.24 on a 5-point Likert-Scale, *SD* = 1.32).

### Measures

#### Job Satisfaction

Job satisfaction was assessed with one item, asking “All in all, how satisfied are you with your work in the coworking space?.” Various studies have shown that the psychometric properties of single-item overall job satisfaction measures are not inferior or even superior to scales using multiple-items (e.g., Wanous et al., 1997; Nagy, 2002). The minimum reliability of a single global item is estimated to be 0.70 (Wanous et al., 1997). The item was anchored to a 5-point Likert-type scale with 1 = “very unsatisfied” to 5 = “very satisfied.”

#### Intent to Leave

For assessing the intent to leave the coworking space, we adopted a scale developed by Lichtenstein et al. (2004). It consists of three items and is used to measure the intention to leave an organization. We adjusted the items to the coworking context by replacing the word “organization” with “coworking space.” An example item is “I will probably look for a new job in the next year,” which was changed to “I will probably look for a new coworking space in the next year.” Answers were given on a 5-point Likert-type scale (1 = “does not apply at all”; 5 = “applies strongly”). Cronbach's alpha was 0.79 in our sample, showing that the changes did not compromise the reliability of this scale.

#### Fit Measures

For a global assessment of PE fit, we used the perceived fit scale by Cable and DeRue (2002). It determines the level of fit from the three perspectives (i.e., NS fit, DA fit and supplementary fit) with three items, each. Like for the outcome scales, the words “organization” or “job” were substituted by “coworking space” or “my work in the coworking space.” An example item was “There is a good fit between what my coworking space offers me and what I am looking for in a coworking space.” (NS fit). Participants rated all items on a 5-point type scale reaching from 1 (“does not apply at all”) to 5 (“applies strongly”). The three-factor structure of the scale has been confirmed (Hinkle and Choi, 2009) and it has proven to be the most reliable tool for global fit assessment (Kristof-Brown and Guay, 2011). In this study, the reliabilities were 0.91 for the NS,.88 for the DA, and 0.92 for the supplementary (PC) fit scale.

To measure NS fit on the domain level (i.e., on the content dimensions community, collaboration, amenities, location, and cost), an indirect approach was used. We assessed how strong the subjective need was (e.g., “The amenities are particularly important to me.”) and the how strong the perceived supply was (e.g., “My coworking space offers good amenities.”). Each statement was rated on a 5-point Likert-type scale ranging from 1 (“does not apply at all”) to 5 (“applies strongly”). With these two values, the indirect-perceived fit was calculated for each content dimension, as explained in the analyses section. This approach allowed us to simultaneously measure NS fit and the respective characteristics of person and environment. In contrast to the global assessment of fit, these insights are especially valuable on the domain level, as they unravel the impact and relationship of the single components on work-related outcomes.

#### Demographic and Employment-Related Variables

As demographic variables, coworkers reported their age and gender. They answered a number of work-related descriptive questions (i.e., their profession, years working in that profession, the industry they would assign their job to, and their contract type). Furthermore, respondents answered a series of questions concerning their coworking space. The items related to this study were duration of the membership, number of weekly work hours spent in the coworking space, average number of people sitting in the same room, who pays for the membership, and if they decided to work there voluntarily. All of the descriptive items were single-item measures with the option to choose a response or a text field to enter their answer.

### Analyses

All analyses were conducted in the statistical computing environment R (R Core Team, 2019). In a first step, we conducted a confirmatory factor analysis (CFA) to validate the factor structure of our measurement model with help of the “Rcmdr” package (Fox, 2005, 2017; Fox and Bouchet-Valat, 2020). For showing the relationship between NS fit and outcomes (H1), a multiple linear regression model was created for each outcome variable, including the mean of NS fit as a predictor, as well as age and gender as control variables. Additionally, we conducted the regression analyses with coworking space related control variables in the model (i.e., years working in their profession, duration of the membership in the coworking space, weekly hours spent in the coworking space, the average number of people in the shared space and voluntariness of their membership) This method enabled us to control for the influence of demographic and coworking space related variables on the outcomes.

H2 and H3 concern the fit of needs and supplies. The calculation of fit from the separate subjective measures has caused a lot of discussion among PE fit researchers (Edwards et al., 2006). The three most common approaches are difference scores, correlations, and polynomial regression (Arthur et al., 2006). However, difference scores and correlations have been criticized repeatedly for their statistical inadequacy, especially for trying to reduce multidimensional fit to a single index (Van Vianen, 2018). Only polynomial regression enables depicting fit as the three-dimensional phenomenon it is. Van Vianen (2018) summarizes three propositions of fit: Fit, as an interactional term, predicts outcomes, rather than person and environment characteristics separately; outcomes are most optimal, when fit is high, independent whether the attributes are low, medium, or high; and the direction of the discrepancy (i.e., lack or excess) does not matter. These propositions can most adequately be tested by analyzing the surface plots resulting from polynomial regression.

The surface plots were created using the “RSA” package (Schönbrodt and Humberg, 2020). They were three-dimensional, with need-level on the x-axis, supply-level on the y-axis and outcomes on the z-axis. They featured a fit line, representing optimal fit (P = E), and a misfit line (P = –E). Fit assumptions were considered supported, when the surface plots met three criteria (e.g., Edwards and Cable, 2009; Van Vianen, 2018). First, the surface should be curved upward (convex) along the misfit line for positive outcomes and curved downward (concave) for negative outcomes. Second, the first principal axis (the ridge of the surface) should run along the fit line. And third, the slope along the fit line should be flat. A polynomial regression model with need- and supply-scores as predictors and job satisfaction or intent to quit as the outcome was tested for each content dimension (H2). For testing H3a the reported need score of community was tested against the need scores of the other four content dimensions using one-sided paralleled *t*-tests. H3b was tested by comparing the models tested for H2.

## Results

### Descriptive Results

Means, standard deviations and intercorrelations of all variables used in the analyses can be found in Table 1. To confirm our measurement model, we compared three models containing all constructs that were measured with more than one item, i.e., intent to leave and the three fit perspectives of the PFS. For considering the model acceptable, we expected the comparative fit index (CFI) to be close to or >0.90, and the root mean square error of approximation (RMSEA) to be close to or <0.08. A CFA supported the fit of the four-factor model with intent to leave as one factor and the three fit perspectives of the PFS as distinct factors, χ^2^(48) = 63.311, *p* = 0.068; CFI = 0.990; RMSEA = 0.042. In contrast, a two-factor model with all PFS items loading on the same factor and the ITQ items loading on one factor did not fit the data well, χ^2^(53) = 689.723, *p* < 0.001; CFI = 0.567; RMSEA = 0.261. A one-factor model with all items loading on the same factor did not fit the data either, χ^2^(54) = 767.634, *p* < 0.001; CFI = 0.514; RMSEA = 0.273.

**Table 1:** Descriptive statistics and correlations.

**Variable**	***M***	***SD***	**1**	**2**	**3**	**4**	**5**	**6**	**7**	**8**	**9**
**Global PE-fit**											
1. NS fit	3.92	0.83									
2. PJ fit	4.02	0.70	0.41^**^								
3. PC fit	3.66	0.94	0.49^**^	0.36^**^							
**Needs**											
4. COM	3.80	1.07	0.24^**^	0.15^*^	0.43^**^						
5. COL	3.28	1.19	0.16^*^	0.08	0.42^**^	0.55^**^					
6. AME	3.91	0.96	−0.02	0.01	−0.03	−0.10	0.04				
7. LOC	4.22	0.87	0.04	0.11	−0.12	0.04	−0.13	0.44^**^			
8. COS	3.47	1.16	0.09	−0.09	0.13	0.05	0.00	0.02	0.07		
**Supplies**											
9. COM	3.90	1.03	0.30^**^	0.11	0.28^**^	0.24^**^	0.31^**^	0.06	−0.01	0.02	
10. COL	3.79	1.04	0.24^**^	0.11	0.31^**^	0.15^*^	0.45^**^	0.05	−0.11	−0.11	0.64^**^
11. AME	3.92	0.95	0.62^**^	0.30^**^	0.40^**^	0.17^*^	0.08	0.10	0.12	0.11	0.13
12. LOC	4.42	0.75	0.24^**^	0.14	0.08	0.06	−0.10	0.22^**^	0.55^**^	0.05	0.16^*^
13. COS	4.12	0.86	0.30^**^	0.29^**^	0.36^**^	0.24^**^	0.20^**^	−0.08	0.06	0.01	0.09
**Outcomes**											
14. SAT	4.27	0.74	0.63^**^	0.42^**^	0.36^**^	0.19^**^	0.13	−0.03	−0.02	−0.01	0.19^**^
15. ITQ	1.92	0.93	−0.53^**^	−0.33^**^	−0.34^**^	−0.18^*^	−0.24^**^	−0.06	0.01	0.20^**^	−0.28^**^
**Control variables**											
16. Age	35.73	10.17	0.09	0.01	0.15^*^	0.03	0.06	0.13	−0.00	0.06	−0.04
17. Gender	1.38	0.51	0.02	0.01	0.02	0.07	0.09	0.04	−0.05	0.08	0.12
18. Years in job	7.90	7.79	0.05	0.06	0.06	−0.02	0.03	0.07	−0.02	−0.07	−0.05
19. Membership length	16.48	16.44	0.11	0.15	0.08	0.02	0.02	−0.15^*^	−0.09	−0.10	−0.00
20. Hours in CWS	28.51	14.38	0.09	−0.00	−0.04	−0.13	−0.05	0.02	0.04	−0.03	−0.03
21. Number of people	7.59	9.01	−0.08	0.02	−0.10	0.01	0.07	0.08	0.09	0.01	0.12
22. Voluntariness	4.24	1.32	0.19^*^	0.19^*^	0.21^**^	0.13	0.09	−0.06	0.00	0.12	0.04
**10**	**11**	**12**	**13**	**14**	**15**	**16**	**17**	**18**	**19**	**20**	**21**
0.20^**^											
0.07	0.35^**^										
0.18^*^	0.21^**^	0.19^**^									
0.19^*^	0.44^**^	0.19^**^	0.33^**^								
−0.31^**^	−0.29^**^	−0.10	−0.33^**^	−0.37^**^							
−0.10	−0.01	−0.08	−0.03	−0.01	−0.19^*^						
0.04	0.00	−0.02	0.01	−0.05	0.01	−0.06					
−0.08	−0.05	−0.10	−0.02	−0.03	−0.16^*^	0.76^**^	−0.04				
0.02	0.03	−0.08	−0.01	0.01	−0.17^*^	0.31^**^	−0.06	0.44^**^			
0.01	0.07	0.12	−0.04	0.11	−0.04	−0.08	−0.15^*^	−0.14	−0.01		
0.11	−0.13	−0.08	0.03	−0.06	0.06	−0.13	0.06	−0.14	0.02	0.11	
0.03	0.18^*^	0.03	0.34^**^	0.22^**^	−0.06	0.19^**^	−0.14	0.24^**^	0.07	−0.19^*^	−0.16^*^

*NS fit, needs-supplies fit; PJ fit, person-job fit; PC fit, person-coworking space fit (supplementary fit); COM, community; COL, collaboration; AME, amenities; LOC, location; COS, cost; SAT, job satisfaction; ITQ, intent to quit; CWS, coworking space*.

* p < 0.05;

** *p < 0.01*.

### Hypothesis Tests

The results of the multiple linear regression analyses can be found in Table 2. When including only the basic control variables age and gender, NS fit was a significant predictor for job satisfaction (a), *B* = 0.58, *SE* = 0.05, *p* < 0.001, and intent to quit (b), *B* = −0.59, *SE* = 0.07, *p* < 0.001. In the case of intent to quit, age was found to also have a small negative effect, *B* = −0.01, *SE* = 0.01, *p* = 0.029. When analyzing the more comprehensive regression model, which also included coworking space related characteristics as control variables, the pattern of our results did not change substantially. Again, NS fit significantly predicted (a) job satisfaction, *B* = 0.55, *SE* = 0.05, *p* < 0.001, and intent to quit, *B* = −0.60, *SE* = 0.07, *p* < 0.001. Of the control variables, only the voluntariness of the coworking space membership was significantly related to job satisfaction, featuring a small positive effect, *B* = 0.07, *SE* = 0.04, *p* = 0.041. The data therefore support H1, showing that NS fit is related to the tested outcomes in the expected way with moderate effect sizes. The higher the coworkers reported their perceived NS fit, the more optimal their work-related outcomes were.

**Table 2:** Regression analysis of NS fit on job satisfaction and intent to quit including control variables.

	**Job satisfaction**			**Intent to quit**		
	***B***	***SE***	***p***	***B***	***SE***	***p***
**Basic model**						
Intercept	2.347	0.275	<0.001	4.650	0.374	<0.001
NS fit	0.578	0.052	<0.001	−0.590	0.071	<0.001
Age	−0.006	0.004	0.188	−0.013	0.006	0.029
Gender	−0.102	0.085	0.230	0.028	0.115	0.809
**Comprehensive model**						
Intercept	2.014	0.342	<0.001	4.382	0.469	<0.001
NS fit	0.554	0.054	<0.001	−0.600	0.074	<0.001
Age	−0.005	0.007	0.461	−0.010	0.009	0.291
Gender	−0.065	0.087	0.461	0.045	0.120	0.709
Years in job	−0.002	0.009	0.845	−0.004	0.013	0.755
Membership length	−0.002	0.003	0.514	0.001	0.004	0.306
Hours in CWS	0.003	0003	0.316	0.001	0.004	0.865
Number of people	−0.001	0.005	0.902	0.001	0.007	0.900
Voluntariness	0.073	0.035	0.041	0.057	0.048	0.241

*NS fit, needs-supplies fit. CWS, coworking space*.

According to H2, the congruence of needs and supplies on the five content dimensions leads to positive work-related outcomes. In order to test if the resulting response surfaces met the three fit assumptions outlined before, four surface tests were conducted. To assure the slope along the fit line is flat, there should be no linear additive effect (a1) and no curvature on the line of congruence (a2). To see if the first principal axis runs along the fit line, it was tested whether the ridge is significantly shifted away from it (a3). Lastly, the expected curvature along the misfit line (a4) was interpreted as concave for significant negative coefficients and convex for significant positive coefficients.

**Figure 2:**
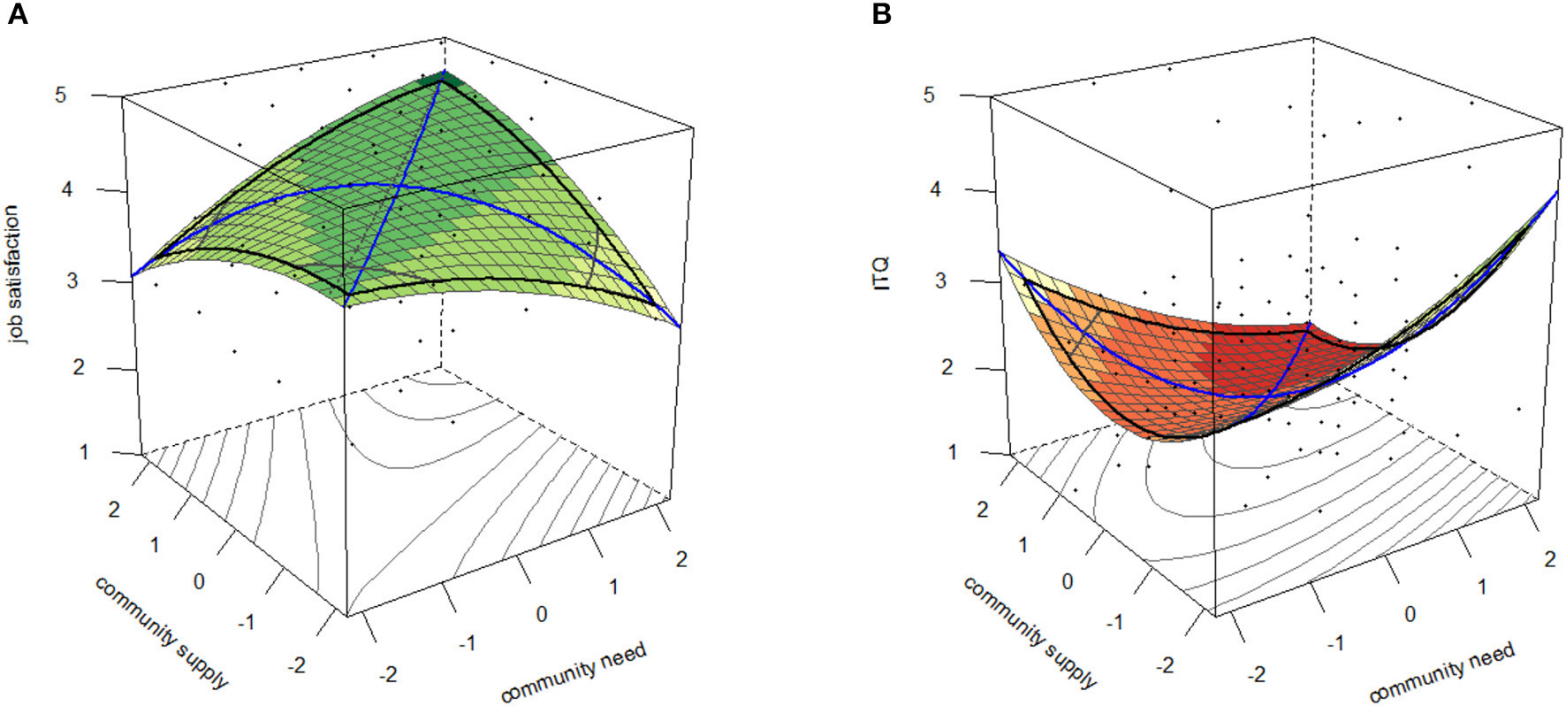
Surface plots of community need and supply on job satisfaction **(A)** and intent to quit **(B)**.

The results of the conducted polynomial regression analyses can be found in Table 3 for job satisfaction and in Table 4 for intent to quit. The surface plots for community fit as a predictor for job satisfaction and intent to quit are presented in Figure 2 for illustration. The model plots for the other content dimensions can be found in the Supplementary Material.

**Table 3:** Polynomial regression analysis results for job satisfaction by content dimension.

	**Community**			**Collaboration**			**Amenities**			**Location**			**Cost**		
	***B***	***SE***	***p***	***B***	***SE***	***p***	***B***	***SE***	***P***	***B***	***SE***	***p***	***B***	***SE***	***p***
**Coefficients**															
C	4.24	0.12	<0.001	4.21	0.10	<0.001	4.15	0.09	<0.001	4.04	0.15	<0.001	3.75	0.14	<0.001
N	0.04	0.05	0.388	−0.05	0.07	0.455	−0.12	0.09	0.175	−0.38	0.18	0.039	0.13	0.09	0.178
S	0.08	0.06	0.230	0.16	0.06	0.009	0.26	0.10	0.011	0.22	0.25	0.395	0.42	0.20	0.033
N^2^	−0.05	0.04	0.143	−0.06	0.04	0.121	−0.02	0.05	0.718	0.11	0.07	0.095	0.05	0.04	0.157
NS	0.14	0.04	<0.001	0.12	0.06	0.092	0.13	0.06	0.021	0.04	0.15	0.793	−0.12	0.06	0.057
S^2^	−0.06	0.04	0.091	−0.03	0.05	0.589	−0.05	0.06	0.339	0.02	0.14	0.888	−0.02	0.09	0.798
*R^2^*	0.11		<0.001	0.05		0.083	0.24		<0.001	0.09		0.004	0.16		<0.001
**Surface tests**															
a_1_	0.12	0.07	0.091	0.11	0.07	0.088	0.13	0.13	0.324	−0.16	0.21	0.460	0.54	0.25	0.029
a_2_	0.03	0.05	0.562	0.02	0.05	0.717	0.06	0.07	0.399	0.17	0.10	0.084	−0.07	0.11	0.485
a_3_	−0.03	0.09	0.717	−0.21	0.11	0.053	−0.38	0.14	0.006	−0.59	0.39	0.124	−0.29	0.18	0.104
a_4_	−0.26	0.08	0.002	−0.20	0.13	0.133	−0.20	0.11	0.050	0.09	0.32	0.770	0.14	0.09	0.110

*C, constant; N, need scores; S, supply scores; a1, linear additive effect on the line of congruence; a2, curvature on the line of congruence; a3, ridge shifted away from the line of congruence; a4, general effect of incongruence*.

**Table 4:** Polynomial regression analysis results for intent to quit by content dimension.

	**Community**			**Collaboration**			**Amenities**			**Location**			**Cost**		
	***B***	***SE***	***p***	***B***	***SE***	***p***	***B***	***SE***	***p***	***B***	***SE***	***p***	***B***	***SE***	***p***
**Coefficients**															
C	1.93	0.13	<0.001	1.96	0.10	<0.001	2.09	0.17	<0.001	1.80	0.19	<0.001	2.17	0.14	<0.001
N	−0.04	0.09	0.662	0.06	0.12	0.591	−0.01	0.13	0.942	0.35	0.19	0.065	0.26	0.09	0.005
S	−0.27	0.11	0.016	−0.41	0.13	0.001	−0.14	0.17	0.405	0.00	0.19	0.999	−0.38	0.09	<0.001
N^2^	0.06	0.05	0.216	0.11	0.05	0.046	0.05	0.07	0.446	0.18	0.12	0.156	0.01	0.04	0.778
NS	−0.17	0.08	0.038	−0.20	0.10	0.039	0.11	0.08	0.182	−0.41	0.20	0.037	−0.09	0.06	0.138
S^2^	0.17	0.07	0.011	0.16	0.07	0.033	−0.01	0.06	0.814	0.06	0.11	0.552	0.04	0.05	0.466
*R^2^*	0.17		<0.001	0.16		<0.001	0.10		0.003	0.06		0.052	0.16		<0.001
**Surface tests**															
a_1_	−0.31	0.13	0.014	−0.35	0.10	<0.001	−0.15	0.24	0.523	0.35	0.27	0.202	−0.12	0.11	0.293
a_2_	0.06	0.07	0.362	0.06	0.06	0.305	0.07	0.11	0.525	−0.17	0.12	0.146	−0.04	0.06	0.499
a_3_	0.23	0.15	0.134	0.47	0.22	0.035	0.13	0.19	0.482	0.35	0.26	0.179	0.63	0.14	<0.001
a_4_	0.40	0.16	0.011	0.47	0.18	0.011	0.15	0.11	0.194	0.65	0.36	0.071	0.16	0.10	0.183

*C, constant; N, need scores; S, supply scores; a1, linear additive effect on the line of congruence; a2, curvature on the line of congruence; a3, ridge shifted away from the line of congruence; a4, general effect of incongruence*.

According to Edwards and Cable (2009) not all the fit assumptions have to be fulfilled to confirm the hypothesized fit. They prioritized the assumptions, rather than seeing them all as binding. First, a general effect of incongruence (a4) is obligatory. Of the tested models to predict job satisfaction, only the community model fulfilled this criterion (*B* = −0.26, *SE* = 0.08, *p* = 0.002). The resulting concave shape of the surface shows that the stronger the discrepancy between need and supply was, the lower NS fit was perceived. For the content dimensions collaboration (*B* = −0.20, *SE* = 0.13, *p* = 0.133), location (*B* = 0.09, *SE* = 0.32, *p* = 0.770) and cost (*B* = 0.14, *SE* = 0.09, *p* = 0.110), this mandatory requirement was not given. For amenities, the effect of incongruence did not meet standard levels of statistical significance (*B* = −0.20, *SE* = 0.11, *p* = 0.050). Therefore, the fit hypothesis had to be rejected for all content dimensions but community. Second, the ridge should run along the fit line (a3), which was the case for the community model (*B* = −0.03, *SE* = 0.09, *p* = 0.717). There was no significant linear additive effect on the fit line (a1; *B* = 0.12, *SE* = 0.07, *p* = 0.091). Further, the analysis showed, that no curvature was present along the fit line (a2; *B* = 0.03, *SE* = 0.05, *p* = 0.562). Therefore, all fit assumptions are sufficiently met. Job satisfaction is highest, when need and supply of community are congruent. The more they differ from each other, the lower the job satisfaction rating.

Among the models predicting the intent to quit of the coworkers, a general effect of incongruence (a4) was found for the content dimensions community (*B* = 0.40, *SE* = 0.16, *p* = 0.011) and collaboration (*B* = 0.47, *SE* = 0.18, *p* = 0.011). As expected for negative outcomes, the surface featured a convex shape. The plots of amenities (*B* = 0.15, *SE* = 0.11, *p* = 0.194), location (*B* = 0.65, *SE* = 0.36, *p* = 0.071) and cost (*B* = 0.16, *SE* = 0.10, *p* = 0.183) did not meet this expectation and could therefore not fulfill the fit assumptions. In contrast to the community model (*B* = 0.23, *SE* = 0.15, *p* = 0.134), the collaboration model failed to fulfill the second requirement (a3), because its ridge deviated from the line of fit (*B* = 0.47, *SE* = 0.22, *p* = 0.035). It is interesting to note, that the negative shift away from the fit line indicates, that lacking collaboration supplies had more detrimental effects on the outcome than an excess of them. Testing the remaining assumptions for the community model, a linear additive effect was found along the fit line (a1; *B* = −0.31, *SE* = 0.13, *p* = 0.014). The negative coefficient indicates that high levels of need and supply predict low levels of intent to quit and vice versa. No significant curvature was detected on the fit line (a2; *B* = 0.06, *SE* = 0.07, *p* = 0.362). This means, that the obligatory fit assumptions are fulfilled. The intent to quit of the respondents was lowest, when fit between needs and supplies was highest. It is of note, however, that the absolute values, in contrast to PE fit assumptions, had an influence, too.

Taken together, the data support H2 for the community content dimension, but not for the others. Community fit is a crucial predictor for job satisfaction and the intent to quit of coworkers. Collaboration, amenities, location and cost, on the other hand, have a relationship to the work-related outcomes that differs from fit.

H3a stated that community has a higher subjective importance than the other need dimensions. The comparisons with one-sided paired *t*-tests showed that community was more important than collaboration, *t*_(179)_ = 6.45, *p* < 0.001; and cost, *t*_(179)_ = 2.91, *p* = 0.002. However, it was not rated more important than amenities, *t*_(180)_ = −0.93, *p* = 0.824; and location, *t*_(179)_ = −4.19, *p* = 1. Hence, the findings did not support H3a.

In H3b the assumption that subjective fit on the community dimension is the best predictor of work-related outcomes was tested. As the results of the polynomial regression analysis revealed, the fit model for community was the only model that withstood empirical testing with regard to the outcomes job satisfaction and intent to quit. Even though the other four models did significantly predict these outcomes as well (except for the collaboration model for satisfaction and the location model for intent to quit), fit effects were only found for community, thereby supporting H3b. What should be noted, is that despite the violation of fit assumptions, the coefficient of determination of the models predicting job satisfaction was highest for amenities (*R*^2^ = 0.24), as compared to the community fit model (*R*^2^ = 0.11), or the collaboration (*R*^2^ = 0.05, n.s.), location (*R*^2^ = 0.09) and cost fit models (*R*^2^ = 0.16). For the intent to quit, the community (*R*^2^ = 0.17), collaboration (*R*^2^ = 0.16), and cost (*R*^2^ = 0.16) models explained more variance than the amenities (*R*^2^ = 0.10) and location (*R*^2^ = 0.06, n.s.) models, respectively.

## Discussion

### Summary and Interpretation of Results

The overall aims of this study were to (1) identify content dimensions of NS fit in coworking spaces and (2) highlight the role of community in the modern knowledge worker society. To this end, we conducted an online study with 181 coworkers from Germany and Austria. Applying a PE fit framework, NS fit on five need dimensions (community, collaboration, amenities, location, and cost) was measured. The relationship of NS fit on these dimensions and work-related outcomes was examined.

Based on the existing theoretical propositions and empirical findings, we hypothesized that NS fit is associated with work-related outcomes and that higher NS fit leads to more optimal outcomes (Hypothesis 1). Consistent with the findings of previous research (for a meta-analysis see Kristof-Brown et al., 2005) it was shown that high NS fit is associated with higher job satisfaction and a lower intent to quit. The first hypothesis has therefore been supported, suggesting that global PE fit assumptions are also valid in non-traditional forms of work.

Zooming into the components of NS fit, we hypothesized that job satisfaction was highest and intent to quit was lowest, if the need for and supply of community, collaboration, amenities, location and cost were congruent (Hypothesis 2). This relationship could only be shown for community. When the need was as high as the amount of supply, work-related outcomes were perceived highest. When the coworking space offered more community or less than desired, the outcomes were perceived lower. The other examined fit dimensions deviated from this pattern. For collaboration and amenities, it was still similar, but maximal job satisfaction and lowest intent to quit were shifted toward exceeding supplies. On the cost dimension, there was no effect of misfit at all. Instead, a good price of the coworking space membership, led to a higher job satisfaction, independent from whether cost was rated important or not. Intent to quit was especially high when the need for adequate cost exceeded the supply. The effect of location fit on job satisfaction and intent to quit was characterized by the extrema and only had a notable misfit effect, when the need for a good location was very high, but the supply very low.

These mixed findings are reflected by the wide range of theoretical conceptualizations of fit in the literature. The definitions form a continuum from very narrow to very broad understandings of fit (Kristof-Brown and Guay, 2011). In this study, we adopted the most restrictive definition of PE fit, namely exact correspondence of needs and supplies. The results contribute to the ongoing discussion if exact correspondence really is an adequate fit conceptualization for all content dimensions. Edwards and Shipp (2007), for instance, expect the effects on the outcome to vary depending on the implications of excess supplies for other needs and for the same need at a later time. Following their line of arguments, outcome scores should only decrease, if excess supplies interfere with the fulfillment of needs on other dimensions. This is the case for community. When the interaction with coworkers goes beyond a person's need for community, it interferes with their need for privacy or a productive work environment and therefore decreases job satisfaction and increases intent to quit. On other content dimensions, however, an excess in supplies does not interfere with other needs. Studies have shown that exceeding supplies can even enhance a positive evaluation of the outcome (e.g., Lambert et al., 2012; Krumm et al., 2013). More collaboration opportunities, for example, can have a carryover effect on business performance, i.e., their excess can be used to fulfill other needs. Whereas, an extremely exceeding collaboration spirit in the coworking space may lead to similar disadvantages as outlined for the community dimension, the cost dimension can basically not overreach expectations. Edwards (1996) would speak of a conservation mechanism, i.e., the money saved due to lower membership costs can be set aside and used at a later point without any negative consequences. Better amenities than needed do not deplete other resources either (as long as cost stays the same), nor do they interfere with them.

Taken together, the suggestions made by Edwards and Shipp (2007), which are in line with contributions by other authors (e.g., Kristof-Brown and Guay, 2011; Van Vianen, 2018), would explain the asymmetric results of the collaboration, amenities and cost dimensions. Consequently, the following question is raised: Is it necessary to give up on the clear theoretical demarcation of fit and adopt a more liberal view instead? This issue will be discussed below. In total, the analysis of H2 did not yield the expected results but can be explained in consistency with past research. It also adds to the literature on differential effects of fit on different content dimensions.

With H3, the crucial role of community in coworking spaces was examined. The momentum of the coworking movement among knowledge workers implied an exceptional importance of community. However, the data suggested that the need for community was not as strong as the more pragmatic aspects amenities and location. Nonetheless, it was rated higher than collaboration and cost (H3a). Drawing a parallel to a traditional psychological theory, may help to explain these findings. According to Maslow hierarchy of needs 1943, basic needs must be fulfilled, before an individual can focus on higher needs. In the work context, such basic needs are a reachable place to work with the required equipment to execute the tasks. If those basics are taken care of, one can strive for higher needs, such as a strong community and good collaboration possibilities. The cost aspect would rather belong to the basic needs because the membership should be affordable in order to work there. However, it seems like this was no salient problem for most coworkers, as they did not consider cost as important as other need dimensions. Further, our results support the assumption that fit on the community dimension is the best work-outcome predictor (H3b), because the community model was the only one where an actual fit effect was observed. What was remarkable, however, was that the models of the other content dimensions could also explain variance in the work-related outcomes, even if it was not fit that drove this effect. According to Cable and Edwards (2004), the environment often dominates fit relationships, because supplies change more frequently than needs and are therefore more salient to people. Looking at the coefficients of the model components, it becomes clear that this was the case in the present sample as well. Our observations therefore blend in with Cable and Edwards's findings and the previous results, which demonstrated that basic (pragmatic) needs have to be fulfilled before considering needs of higher (social) nature.

### Limitations and Future Research

As with any study, there are certain limitations that hold potentially useful avenues for future research. The first set of limitations concerns general aspects of the study design. The correlational research design does not allow a verification of the causal relationship between fit and the outcomes. Additionally, it is assumed that perceptions of fit are relative. Potential comparison processes between past, present and future fit may be an influential factor (Edwards et al., 1998). Fit research including the time dimension is still scarce and should be addressed in future studies with a longitudinal design. Also, the sample size of the study, though sufficient for the current analyses, may be too small to derive generalizable conclusions from it.

A second limitation is that dependent and independent variables were assessed with self-report survey methods. Consequently, the problem of common-method bias cannot be ruled out (Podsakoff and Organ, 1986). We followed the recommendations for fit measurement via subjective reports by Cable and Judge (1997). Future studies should therefore consider using a different outcome measure, e.g., actual turnover behavior instead of the self-reported intent to quit.

Going into further detail, some limitations regarding the measurement of PE fit became apparent. The results of this study suggest that the measurement of fit as exact correspondence was not optimal. Fit hypotheses on most dimensions had to be rejected due to the strict understanding of fit. As Kristof-Brown and Guay (2011) have noted, conceptualizations of fit may vary. Instead of exact correspondence, some authors describe fit as a general compatibility of person and environment (Dawis and Lofquist, 1984). This understanding of fit, however, is very vague and bears the danger, that the concept is defined by its consequences (Kristof-Brown and Guay, 2011). Between these two extrema lies the definition of fit as commensurate compatibility. This concept requires a relationship between person and environment on the same dimension, which can vary within a wider range. Kristof-Brown and Guay (2011) conclude that the selection of the measurement type should depend on the fit concept. If exact correspondence is examined, person and environment should be assessed separately. This was the procedure for the needs and supplies measures in the present study. It was chosen because it has several strengths. For instance, it allows detailed insights into the single variables and a separate statistical treatment of them. Especially since this was the first study to examine NS fit in coworking spaces, we opted for an approach that enabled us to take a closer look at the respective components of the model. However, if compatibility is examined, direct measures of perceived fit are considered more appropriate. We adopted this approach for the global fit measures. Based on the results, a compatibility definition of fit appears more adequate for the needs and supplies as well. Hence, future studies on NS fit should consider using direct measures of fit when it comes to the prediction of outcomes.

There is another limitation related to the indirect measurement of fit. Edwards et al. (2006) and Van Vuuren et al. (2007) have found that the links between direct-perceived and indirect-perceived fit are surprisingly weak. This indicates that they do not necessarily reflect the same psychological construct. Accordingly, we treated NS fit on the need domains and global NS fit independently. A potential explanation for the weak links between the measurement types are comparative judgement mechanisms within the individual that act as a mediator between separate perceptions of person and environment and fit perceptions (Cranny et al., 1992; Edwards et al., 2006). Other studies suggest that global NS fit perceptions are derived from fit perceptions on the domain level. For instance, Travaglianti et al. (2017) propose a model, in which the effect of domain-level NS fit on behavioral outcomes is mediated by global NS fit. They use information integration theory (Anderson, 1962) as an explanatory framework. Yet, research on any of these mechanisms is scarce and deserves further investigation. In our study, we only included one measurement type (direct- or indirect-perceived) per fit level (domain or global). To compare the constructs and gain further knowledge about fit mechanisms, however, it would be necessary to include both measures for each of them. Due to the different measurements of fit and the cross-sectional study design, it was not possible to investigate any mediation effects. Future studies should take these relationships further into account.

Furthermore, there are potential moderators of fit that were not considered in this study. The first moderator is the importance of the need dimension (Edwards and Shipp, 2007). For example, Cooper and Kurland (2002) found that remote workers only feel isolated, if they value activities like interpersonal networking, informal learning, and mentoring. As our results indicate, not all needs were equally important to the coworking space users. However, the moderating role of the importance of the respective fit dimensions on the outcomes could not be investigated, because importance and need level were confounded in the design of the questionnaire. Instead of measuring importance and need level separately, respondents were asked how important each need dimension was to them. This answer was considered to reflect their need strength. It is important for future studies to avoid such hybrid items (Edwards and Shipp, 2007).

A second potential moderator is age. For instance, Krumm et al. (2013) showed, that age acts as a moderator of the effect of NS fit on job satisfaction. The higher the age, the stronger the negative impact of NS misfit was. Analyses in this study did not involve age as a predictor in order to reduce complexity. It should also be noted that past research found age to be a significant predictor of the investigated outcomes (see Rhodes, 1983 for a review). This can be considered a shortcoming of the present study.

What could be extended in future studies concerning PE fit in coworking spaces are the fit perspectives. Most researchers distinguish between NS fit, DA fit and supplementary fit. Among these fit perspectives, we focused on NS fit because it seemed (and proved) to have the highest relevance for coworking space users. However, other distinctions of PE fit exist, that can be of special importance in coworking spaces. The fit between an individual and the group of coworkers, for example, would be an interesting pathway for future research to follow. Especially, regarding the results of this study, which indicate that people have specific ideas about the community when joining a coworking space.

The last limitation that should be mentioned concerns the conceptualization of the fit items. Spinuzzi et al. (2018) recently criticized, that the terms community and collaboration are too imprecise to investigate them properly. The other need dimensions could also bear the risk of ambiguity regarding their relationship with different coworking types. For example, amenities could refer to equipment necessary for working (pragmatic), but also to leisure amenities, such as table soccer or coffee bars (social). We only used one item for each content dimension to set a starting point for further need research in coworking spaces. It is essential for future studies to elaborate on the subdimensions of broader need categories. This would allow the use of factor models to investigate user profiles more effectively.

### Practical Implications and Conclusion

The findings of this study have practical implications for both, coworking space operators and users. Deskmag (2019) reported, that the global number one challenge for coworking spaces is attracting new members. Especially in bigger cities the competition is increasing as more coworking spaces are opening each year. Hosts come up with various ideas to tackle this issue. Examples are events, expensive advertising, and “free coworking days” (Deskmag, 2017). Yet, one of the simplest and most economic strategies for coworking spaces is finding a niche in the market and specialize in a certain target group (Benedikt, 2019; Meunier, 2019). This is where the present study can be of help. An evident approach would be to specialize in a certain profession, e.g., IT professionals or entrepreneurs. However, an advantage of coworking spaces, that is highly valued by users, is the diversity and interdisciplinarity among the coworkers (Fuzi, 2015; Merkel, 2015; Deskmag, 2017). By setting the benchmark to more abstract properties, such as the strength of the community, this quality does not get lost. Supporting this suggestion, the results of this study imply that people differ in their need for community and that the community supplied by the coworking space should correspond to the desired amount. This way it will lead to higher satisfaction and lower intentions to change the workplace. In conclusion, the degree to which community is offered in a coworking space can be used as an additional argument within a broad marketing strategy.

As Judge and Cable (1997) have noted, perceived fit with an organization is a key factor when deciding to apply for a job or accepting an offer. In a similar manner, potential members will decide for a coworking space based on their perceived fit with it, and especially with the community. This should be considered when developing the image and public relations strategies of the coworking space. Summarizing the implications for operators, the results do not only specify factors for satisfying or maintaining users, but also help to address their most important difficulty: attracting new members.

Users will obviously profit from these insights as well. Any efforts the hosts put into specializing in certain needs will make it easier for the target audience to see the difference and personal value of the respective coworking spaces. Knowing not only what they want, but also what the coworking space offers, will help them to pick a corresponding workplace and profit from it. In about half of the coworking spaces worldwide an increasing turnover rate has been observed (Deskmag, 2019). This indicates that users did not find a space that met their expectations and kept looking for a better match among the growing number of alternatives. It is certainly not an easy task for hosts to please each member. While 24% report a lack of interaction with others to be their main problem, a similarly high proportion complains about a lack of privacy (Deskmag, 2017). This notion reflects how important fit is and how a clear characterization of the coworking space concept would be helpful for both, operators, and users.

In conclusion, the results of this study contribute to PE fit literature and provide initial empirical insights into the recently booming phenomenon of coworking spaces. With a sample of German and Austrian coworkers, this study showed that users of coworking spaces differ in their needs. While basic needs, such as amenities or the location of the coworking space were important to most participants, social needs differed between persons. The results suggest a pragmatic and a social focus of coworking. Despite potential methodological limitations, this study provides evidence that PE fit, especially NS fit, is associated with work-related outcomes. However, it prompts questions about the adequate measurement of NS fit. Further research is now needed that explores the psychological mechanisms and the increasingly important role of community fit for knowledge workers in alternative work arrangements.

## Data Availability Statement

The datasets generated for this study can be found in online repositories. The names of the repository/repositories and accession number(s) can be found below: https://osf.io/2sn8z/?view_only=5cde231c095941068a7e2a07954d2916.

## Ethics Statement

Ethical review and approval was not required for the study on human participants in accordance with the local legislation and institutional requirements. The patients/participants provided their written informed consent to participate in this study.

## Author Contributions

EL and HZ conceived and designed the study. EL analyzed the data, interpreted the results, and drafted the article. All authors contributed to the article and approved the submitted version.

## Conflict of Interest

The authors declare that the research was conducted in the absence of any commercial or financial relationships that could be construed as a potential conflict of interest.
