# Supplementary figures and images for: Do We Have a Match? Assessing the Role of Community in Coworking Spaces Based on a Person-Environment Fit Framework
Source: Front Psychol. 2021 Feb 9;12:620794. doi: 10.3389/fpsyg.2021.620794 (PMC7900191; doi:10.3389/fpsyg.2021.620794)

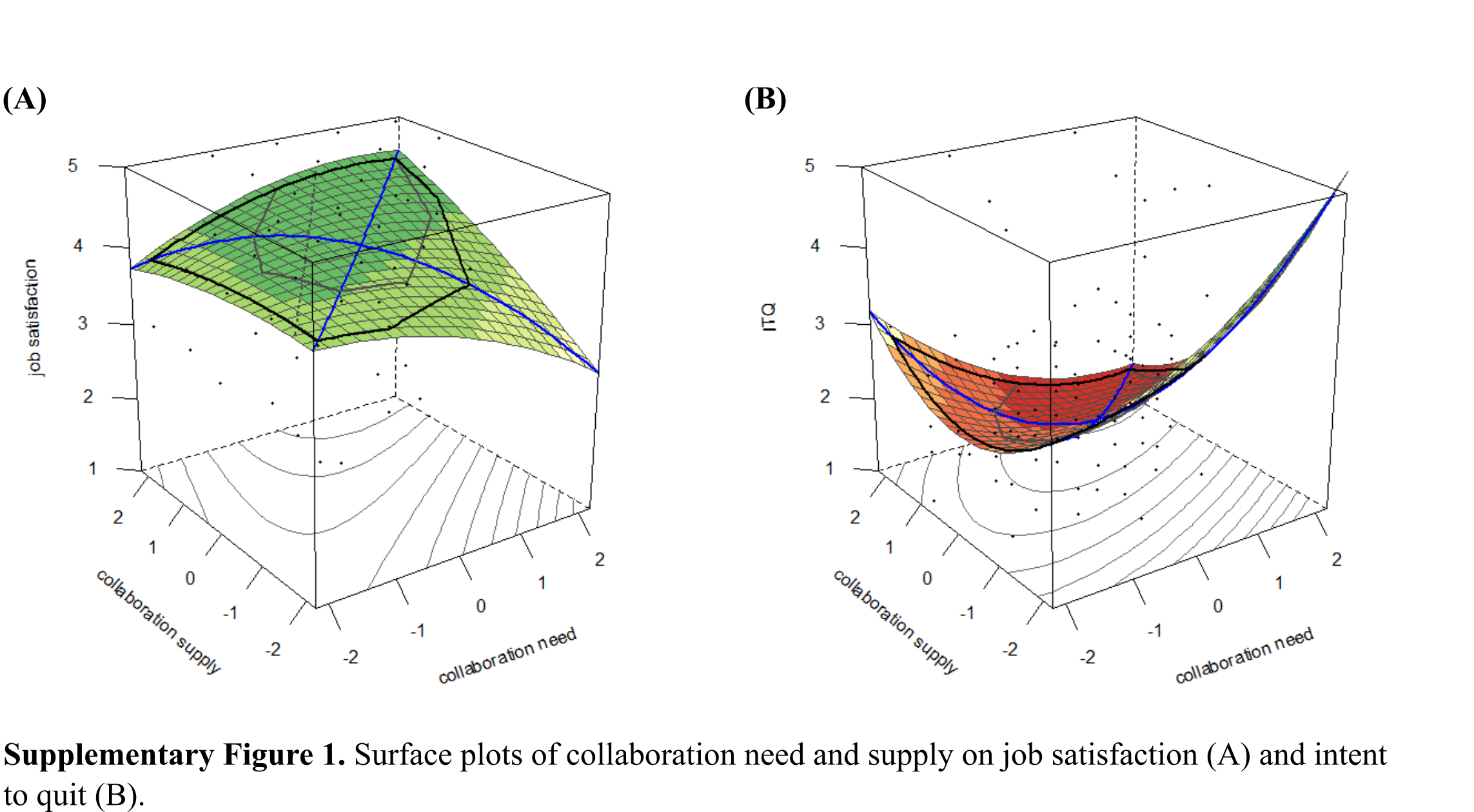

Supplement: Supplementary file 1 [file Image_1.TIF]

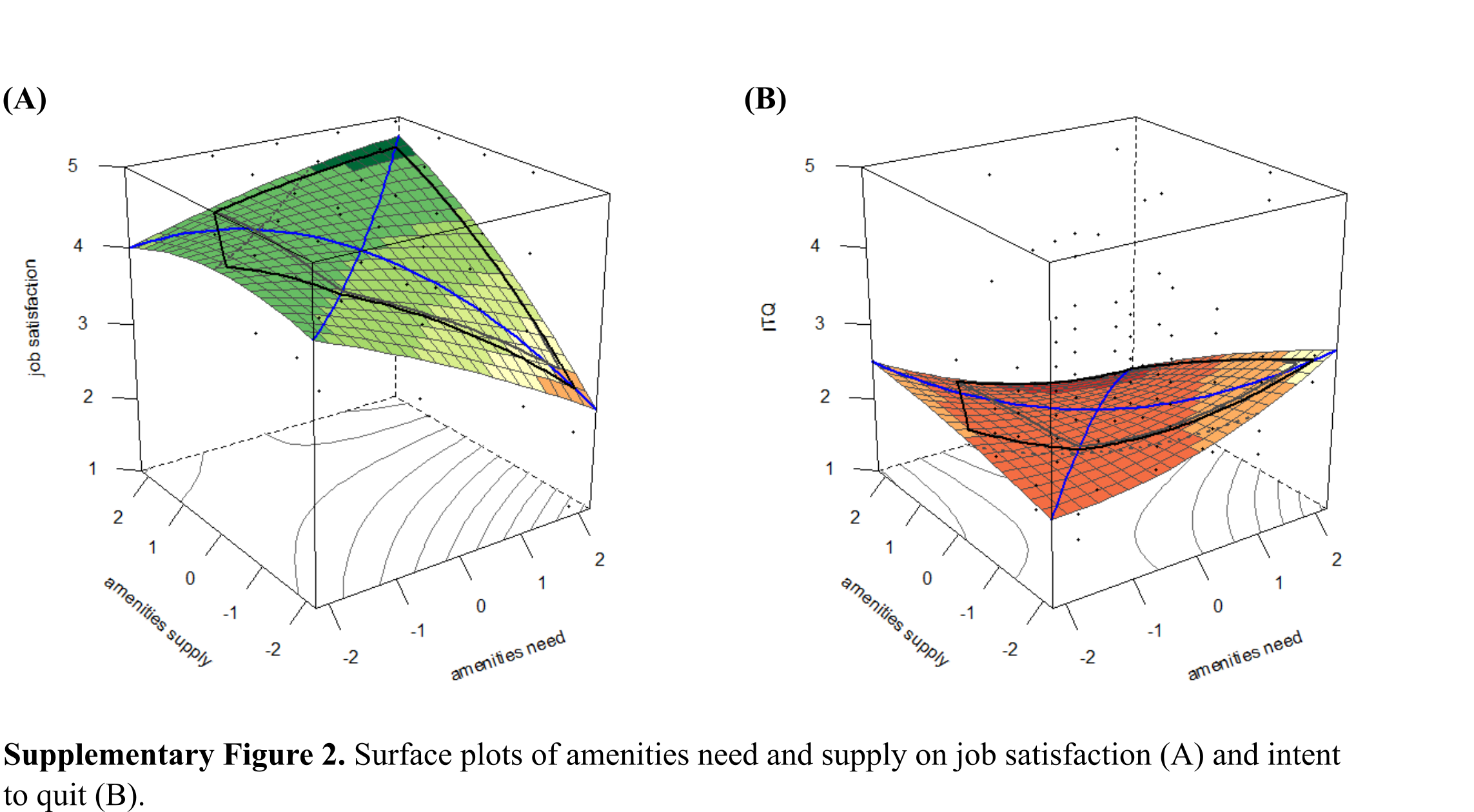

Supplement: Supplementary file 2 [file Image_2.TIF]

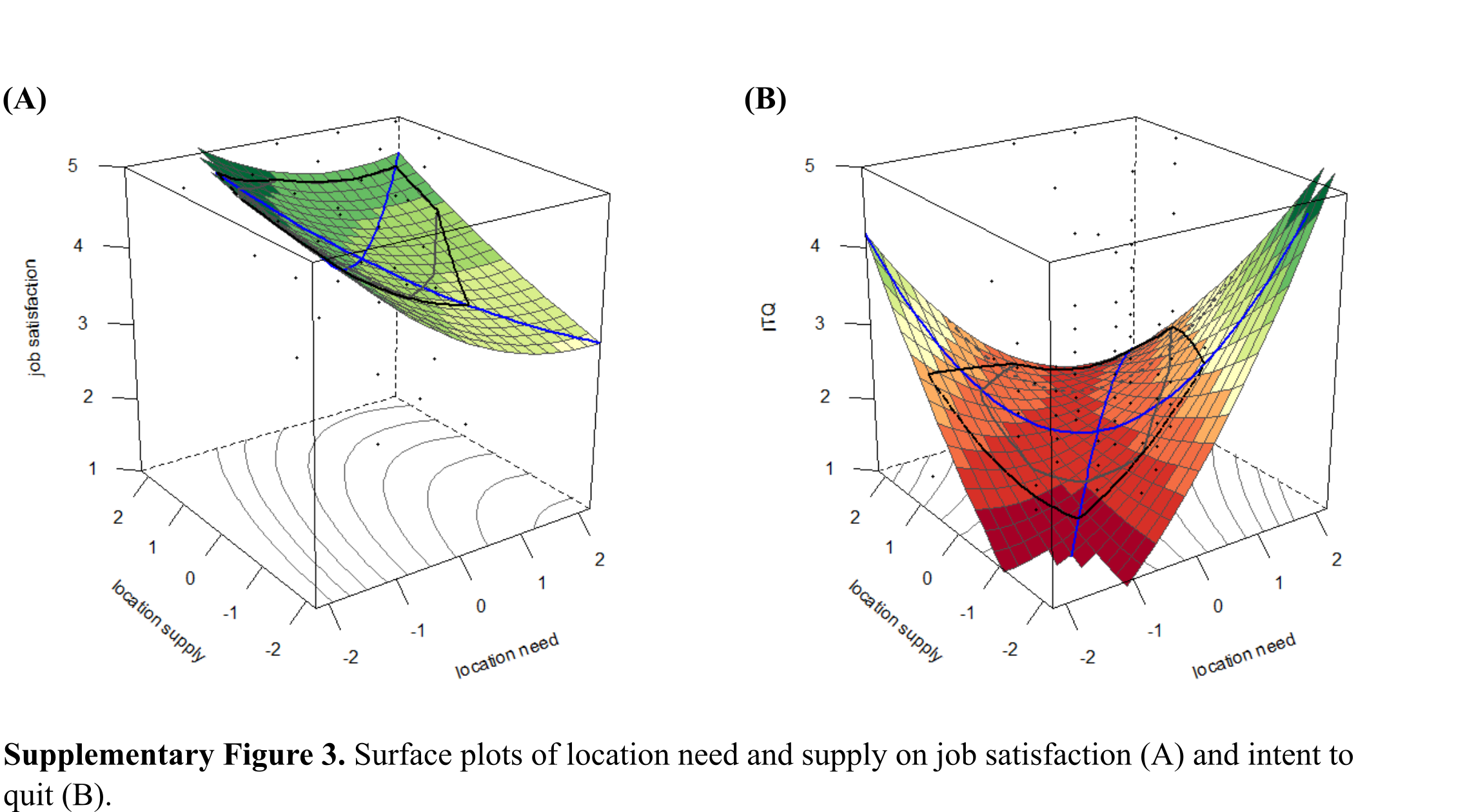

Supplement: Supplementary file 3 [file Image_3.TIF]

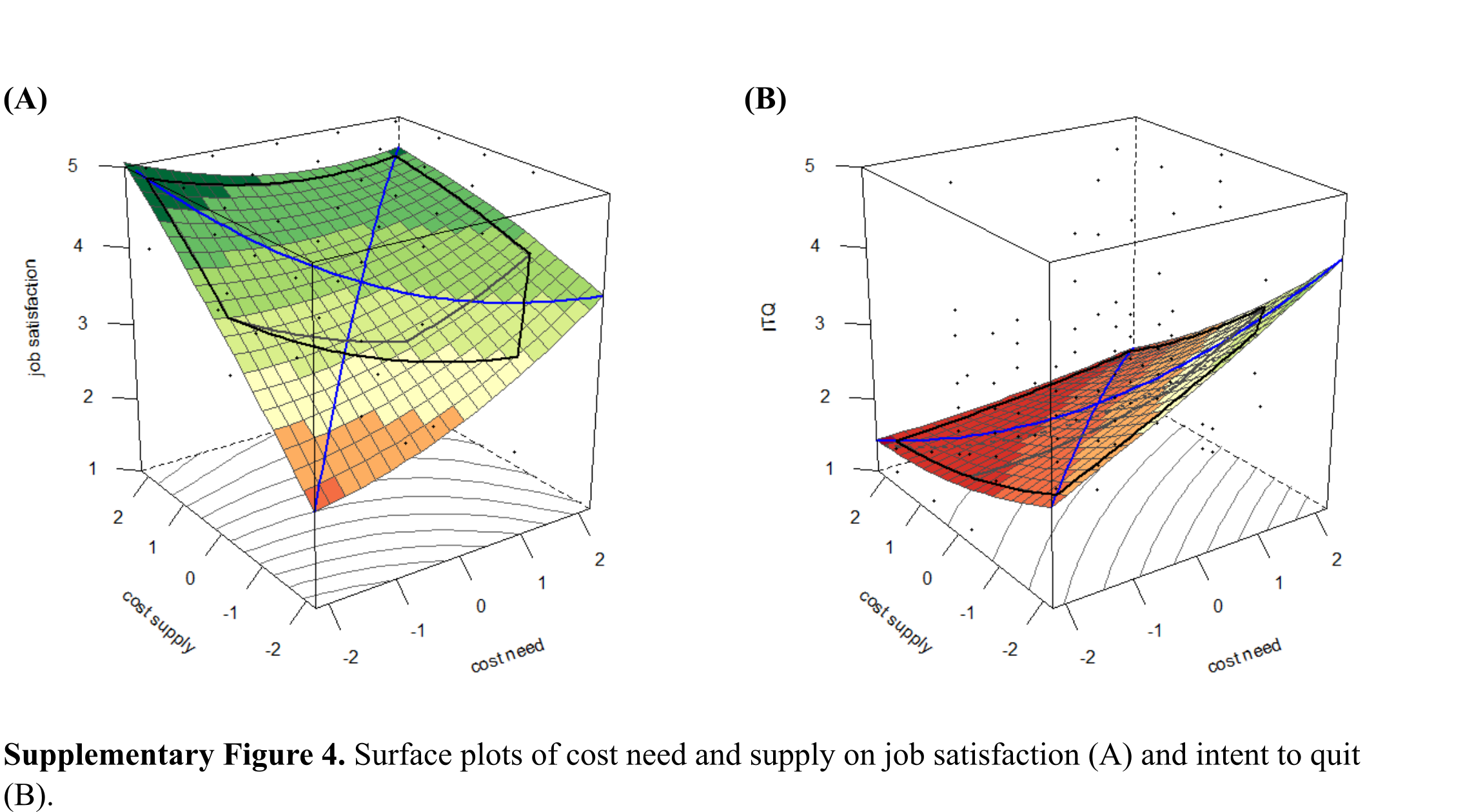

Supplement: Supplementary file 4 [file Image_4.TIF]
